# Vitamin-D Deficiency & Depression: Is There an Association? Average Data From Gulf Cooperation Council Countries

**DOI:** 10.1192/bjo.2024.177

**Published:** 2024-08-01

**Authors:** Shamil Wanigaratne, Amar Ahamd, Fatme Al Anouti, Mitha Al Balushi, Syed Javaid

**Affiliations:** 1 Kings College, London, United Kingdom; 2 Public Health Research Center, New York University-Abu Dhabi, Abu Dhabi, UAE; 3 Zayed University, Abu Dhabi, UAE; 4 Institute of Public Health, College of Medicine and Health Sciences, Abu Dhabi, UAE; 5 Department of Psychiatry and Behavioral Sciences, College of Medicine and Health Sciences, Abu Dhabi, UAE

## Abstract

**Aims:**

The evidence for the association between vitamin-D deficiency and depression, although equivocal, has been established in several populations in different countries and supported by meta-analytical studies^1^. Much of the evidence for this comes from Western countries^2^. Similarly, the evidence for the benefits of supplementation, although shown, also comes from similar populations and is equivocal^3^. Need for data from different populations and for randomized controlled trials to establish causality is stressed by most researchers. This study aims for presentation reviews of the association between vitamin-D and depression in the GCC, using the publicly available data of Our World in Data.

**Methods:**

The statistical analysis used median prevalence depressive disorders data (from 1990–2019) in the GCC countries (both sex and age-standardized (%)), which was downloaded from Our World in Data and was last updated on August 28, 2022. Vitamin D deficiency data were collected through a literature review search using PubMed and Google Scholar. A linear regression model was performed with the median prevalence of depressive disorders data as an outcome. The prevalence of vitamin-D deficiency, population median age and the interaction term between prevalence of vitamin-D deficiency and population median age were used as predictors. The effects of prevalence of depressive disorders both sex age standardized (AS) percentage (%) were estimated with 95% confidence interval (95% CI) using bootstrap covariance matrix estimator. Fitted model's likelihood ratio chi-square (LR χ^2^) test with corresponding p-value was computed and reported.

**Results:**

A positive association was observed between the median prevalence of depressive disorders and the prevalence of vitamin-D deficiency, adjusted for population median age, were observed (LR χ^2^ p-value = 0.005) and adjusted R^2^ = 0.706.

**Conclusion:**

Prevalence of depressive disorders was associated with prevalence of vitamin D deficiency among the population of GCC countries. Future randomized control trials on Vitamin D supplementation are needed to confirm these observations.

